# MicroRNA-146a Is Upregulated by and Negatively Regulates TLR2 Signaling

**DOI:** 10.1371/journal.pone.0062232

**Published:** 2013-04-29

**Authors:** Edel M. Quinn, Jiang Huai Wang, Grace O’Callaghan, H. Paul Redmond

**Affiliations:** 1 Department of Academic Surgery, University College Cork, Cork University Hospital, Cork, Ireland; 2 Department of Medicine, University College Cork, Cork University Hospital, Cork, Ireland; Charité-University Medicine Berlin, Germany

## Abstract

TLR signaling is a crucial component of the innate immune response to infection. MicroRNAs (miRNAs) have been shown to be upregulated during TLR signaling. Specifically, microRNA-146a (miR-146a) plays a key role in endotoxin tolerance by downregulating interleukin-1 receptor-associated kinase 1 (IRAK-1). The aim of this study was to assess the role of miR-146a in the TLR2 signaling and development of bacterial lipoprotein (BLP) self-tolerance and cross-tolerance to bacteria. Expression of miR-146a increased in a dose- and time-dependent manner in BLP-stimulated human THP-1 promonocytic cells. In BLP-tolerised cells miR-146a was even further upregulated in response to BLP re-stimulation (*p*<0.001). Re-stimulation of BLP-tolerised cells with heat-killed gram-negative *Salmonella typhimurium* (*S. typhimurium*), but not gram-positive *Staphylococcus aureus* (*S. aureus*), led to significant overexpression of miR-146a (*p*<0.05). Transfection of naive cells with a miR-146a mimic substantially suppressed TNF-α production (*p*<0.05). Furthermore, overexpression of miR-146a resulted in strong reduction in IRAK-1 and phosphorylated IκBα expression in naive and *S. typhimurium*-stimulated THP-1 cells. Collectively, miR-146a is upregulated in response to BLP and bacterial stimulation in both naive and BLP-tolerised cells. Overexpression of miR-146a induces a state analogous to tolerance in BLP-stimulated cells and therefore may represent a future target for exogenous modulation of tolerance during microbial infection and sepsis.

## Introduction

Sepsis is the leading cause of death in patients who are critically ill [1] and the annual incidence of sepsis continues to increase [2]. Sepsis is the systemic inflammatory response to infection and the initial host response to infection is mediated via activation of the innate immune system [3]. The cells of the innate immune system express pattern recognition receptors (PRRs), such as Toll-like receptors (TLRs) which recognize and transduce signals on contact with bacterial components [3]. The immune response is initiated following recognition of these structural components of bacteria, called pathogen-associated molecular patterns (PAMPs), such as lipopolysaccharide (LPS) or endotoxin in gram-negative bacteria and peptidoglycan (PGN) in gram-positive bacteria [4]. There are 10 functional TLRs in humans and each has a distinct function in terms of PAMP recognition and immune responses [5]. TLR2 responds to PGN, lipoteichoic acid (LTA) and bacterial lipoprotein (BLP), whereas TLR4 responds to LPS [5], [6]. TLR2 signals via the myeloid differentiation factor 88 (MyD88)-dependent signaling pathway [7].

Pre-exposure of monocytes/macrophages (*in vitro*) or the host (*in vivo*) to LPS or endotoxin induces a transient state of cellular hyporesponsiveness to a secondary LPS challenge with diminished production of proinflammatory cytokines, thereby conferring protection against LPS-induced lethality and resulting in a significant survival advantage [8]–[10]. This phenomenon is well established and termed endotoxin tolerance. Furthermore, emerging evidence has revealed that modulation of the TLR4-mediated signal transduction pathway is involved in the development of endotoxin tolerance [9], [10]. For example, down-regulated cell-surface expression of TLR4, reduced interleukin-1 receptor-associated kinase 1 (IRAK-1) expression and myeloid differentiation factor 88 (MyD88)-IRAK immunocomplex formation, and attenuated nuclear factor (NF)-κB activation and mitogen-activated protein kinase (MAPK) phosphorylation are all considered as significant markers for endotoxin tolerance. Therefore, endotoxin tolerance may represent a protective mechanism, whose primary function is to prevent excessive inflammatory response induced by overactivation of the TLR4 signaling pathway. It has been shown that endotoxin tolerance occurs in several disease settings including sepsis, trauma, surgery and pancreatitis [9]; however, acquisition of endotoxin tolerance may contribute to an increased incidence of secondary bacterial infection in hospitalized patients due to development of an immunesuppressed state [9], [10].

Tolerance to components of gram-positive bacterial cell wall such as BLP and LTA also occurs [11], although this has been a lesser studied phenomenon [12]. BLP tolerance is associated with a reduction in levels of tumor necrosis factor-α (TNF-α) and interleukin (IL)-6 after subsequent BLP stimulus [11], via suppressed NF-κB activation [13]. Cross-tolerance of endotoxin-primed cells to other TLR ligands has also been described [14], following endotoxin exposure cells are hyporesponsive to stimulation with other TLR ligands as well as hyporesponsive to further endotoxin stimulation. The clinical significance of tolerance development and potential exogenous modulation of tolerance in human sepsis is as yet undetermined.

MicroRNAs (miRNAs) are a class of evolutionarily conserved small RNA [15], the first discovered members of which were *lin-4* and *let-7* [16]–[18]. Since then hundreds of miRNAs have been identified in plants, animals and viruses [19], with more than 700 miRNA genes identified in the human genome [20]. miRNAs bind to the 3′ untranslated (UTR) region of target messenger RNA to downregulate gene expression [16]–[18]. miRNAs are transcribed by RNA polymerase II or RNA polymerase III to generate a stem-loop, containing primary miRNA [19]. This primary miRNA is processed by the RNase III enzyme, Drosha, to produce precursor miRNA, which is then cleaved to produce the mature miRNA by another RNase III enzyme, Dicer [19]. The miRNA is incorporated into the RNA-induced silencing complex (RISC) and guides the RISC to its RNA targets by base-pairing interactions [19]. miRNAs regulate target mRNA at the posttranscriptional level either by mRNA degradation or translational repression [21], [22].

miRNAs have been found to have diverse roles, including roles in immune responses [15], [23]–[25]. Taganov et al. [15] first demonstrated that the human miRNAs miR-146a/b, miR-132 and miR-155 are upregulated in response to LPS stimulation of the human promonocyte THP-1 cell line. Nahid et al. [23] subsequently confirmed miR-146a upregulation in response to LPS in THP-1 cells, additionally showing that levels of miR-146a continue to rise with ongoing exposure to LPS beyond 24 h and that miR-146a expression is LPS dose-dependent. Nahid et al. [23] further went on to describe a role for miR-146a in endotoxin tolerance development via negative feedback on tumor necrosis factor receptor-activated factor 6 (TRAF6) and IRAK-1. A similar response has been seen in miR-146a-transfected cells exposed to subsequent stimulation with BLP [26].

The aim of this study was to examine the putative role of miR-146a as a contributor to the development of BLP tolerance and to further assess for a role for miR-146a in BLP cross-tolerance to both gram-positive and gram-negative bacteria. We report here that miR-146a is upregulated in response to either BLP or bacterial stimulation in both naive and BLP-tolerised human THP-1 cells and that miR-146a negatively regulates the TLR2 signaling pathway in BLP-induced self-tolerance and cross-tolerance to gram-negative bacteria.

## Materials and Methods

### Reagents

RPMI-1640 supplemented with 10% heat-inactivated fetal calf serum (FCS), penicillin (100 units/ml), streptomycin sulphate (100 µg/ml) and glutamine (2 mM) was used as the cell culture medium for all experiments except transfection experiments where RPMI-1640 without added FCS and antibiotics was used. All medium and reagents used for cell cultures were purchased from Invitrogen Life Technologies (Paisley, Scotland, U.K.). Synthetic bacterial lipoprotein (Pam3-Cys-Ser-Lys4-OH) that was endotoxin-free as confirmed by the Limulus amebocyte lysate assay (Charles River Endosafe, Charleston, SC) was obtained from EMC Microcollections (Tubingen, Germany) and reconstituted to the necessary concentrations using sterile phosphate buffered saline (PBS) (Invitrogen Life Technologies).

### Cell Cultures and Induction of Tolerance

The undifferentiated human promonocytic THP-1 cell line obtained from the American Type Culture Collection (ATCC, Manassas, VA) was cultured in complete RPMI 1640 at 37°C in humidified 5% CO_2_. For tolerance experiments, cells at a density of 1×10^6^ or 5×10^5^ cells/ml were plated in 24-well plates (Falcon, Lincoln Park, NJ) and incubated with 100 ng/ml BLP for 18 h to induce tolerance as demonstrated previously [13], [26], [27] and then washed twice with PBS prior to being stimulated with BLP or heat-killed bacteria or left in culture medium alone as a control. Dose-response effects were established by using different tolerising doses of BLP; in these experiments cells were incubated with either 10, 100 or 1,000 ng/ml BLP for 18 h prior to re-stimulation.

### Preparation of Heat-killed Bacteria

Gram-positive *Staphylococcus aureus* (*S. aureus*) and gram-negative *Salmonella typhimurium* (*S. typhimurium*) were obtained from ATCC and the National University of Ireland Culture Collection, respectively. Bacteria were cultured at 37°C in trypticase soy broth (Merck, Darmstadt, Germany), harvested at the midlogarithmic growth phase, washed twice and resuspended in PBS. The concentration of resuspended bacteria was determined and adjusted spectrophotometrically at 550 nm. Different concentrations were made at 2×10^7^ CFU/ml for *S. aureus* and 2×10^6^ CFU/ml for *S. typhimurium*. Aliquots of 1 ml of each were heat-killed at 95°C for 20 min before being used immediately or stored at -80°C until further use.

### RNA Extraction, Reverse Transcription and Real-time PCR

RNA was extracted from THP-1 cells using the miRNeasy Mini Kit (Qiagen, Crawley, U.K.) as per manufacturer’s instructions. Extracted RNA was diluted to concentrations of 1 ng/µl using nuclease-free water (Applied Biosystems, Carlsbad, CA). cDNA synthesis was performed using the TaqMan MicroRNA Reverse Transcriptase kit (Applied Biosystems) and specific primers from TaqMan microRNA assays (Applied Biosystems) for miR-146a using 5 µl (5 ng) of RNA for each reverse transcriptase reaction, as per manufacturer’s instructions. Real-time quantitative PCR (qRT-PCR) was performed using TaqMan microRNA assays (Applied Biosystems) following the manufacturer’s protocol. miR-146a expression levels were normalized to the stably expressed endogenous small nucleolar RNA control RNU44 (Applied Biosystems) as validated previously [23]. Expression levels of miR-146a were calculated using the 2^−ΔΔCt^ method [28].

### Cell Transfection

Prior to transfection, 500 µl of THP-1 cells at 5×10^5^ cells/ml were plated into each well of 24-well plates with serum- and antibiotic-free RPMI-1640 culture medium, and incubated at 37°C with 5% CO_2_ for 12 h. Cells were transfected with 40 nM of either miR-146a mimic or miRNA negative control (Ambion, Carlsbad, CA) using 1 µl lipofectamine 2000 (Invitrogen Life Technologies). Twelve hours after transfection, cells were further stimulated with either complete culture medium, 1,000 ng/ml BLP, 1×10^6^ CFU/ml *S. typhimurium* or 1×10^7^ CFU/ml *S. aureus* for 6 h.

### Measurement of TNF-α, IRAK-1 and Phosphorylated IκBα

TNF-α concentrations in cell-free supernatants from different experiments were assessed by ELISA (eBioscience, Hatfield, U.K.) as per manufacturer’s instructions.

Naive, BLP-tolerised and miR-146a mimic-transfected THP-1 cells were stimulated with 1×10^6^ CFU/ml *S typhimurium* for various time periods, washed with ice-cold PBS and lysed on ice in cell lysis buffer (Cell Signaling Technology, Beverly, MA) supplemented with 1 mM phenylmethylsulfonyl fluoride and protease inhibitor cocktail (Roche, Indianapolis, IN). The resultant lysates were centrifuged and supernatants containing the cytoplasmic proteins were collected. Protein concentration was measured using a micro bicinchoninic acid protein assay (Pierce, Rockford, IL). Equal amounts of protein extracts were separated on a 10% SDS-polyacrylamide gel and trans-blotted onto polyvinylidene difluoride membranes (Schleicher & Schuell, Dassel, Germany). Membranes were blocked for 1 h at room temperature with 5% nonfat milk in 0.1% PBS/Tween-20 and probed overnight at 4°C with the appropriate primary Ab IRAK-1 (Cell Signaling Technology) or phospho-IκBα (Cell Signaling Technology). Blots were then incubated with the appropriate HRP-conjugated secondary Ab (Dako, Cambridge, U.K.) at room temperature for 1 h, developed with the enhanced chemiluminescence system (Pierce) and captured with Scion image system (Scion Inc, Frederick, MD).

### Statistical Analysis

Data are expressed as mean ± standard error of the mean. Statistical analysis was performed using Student’s t test for normally distributed data and Mann-Whitney-U test for non-parametric data. Correlations between TNF-α and miR-146a in response to BLP stimulation were assessed using the Pearson correlation coefficient test. The *p* values <0.05 were considered statistically significant.

## Results

### miR-146a is Upregulated by Activation of the TLR2 Signaling

**Figure 1:**
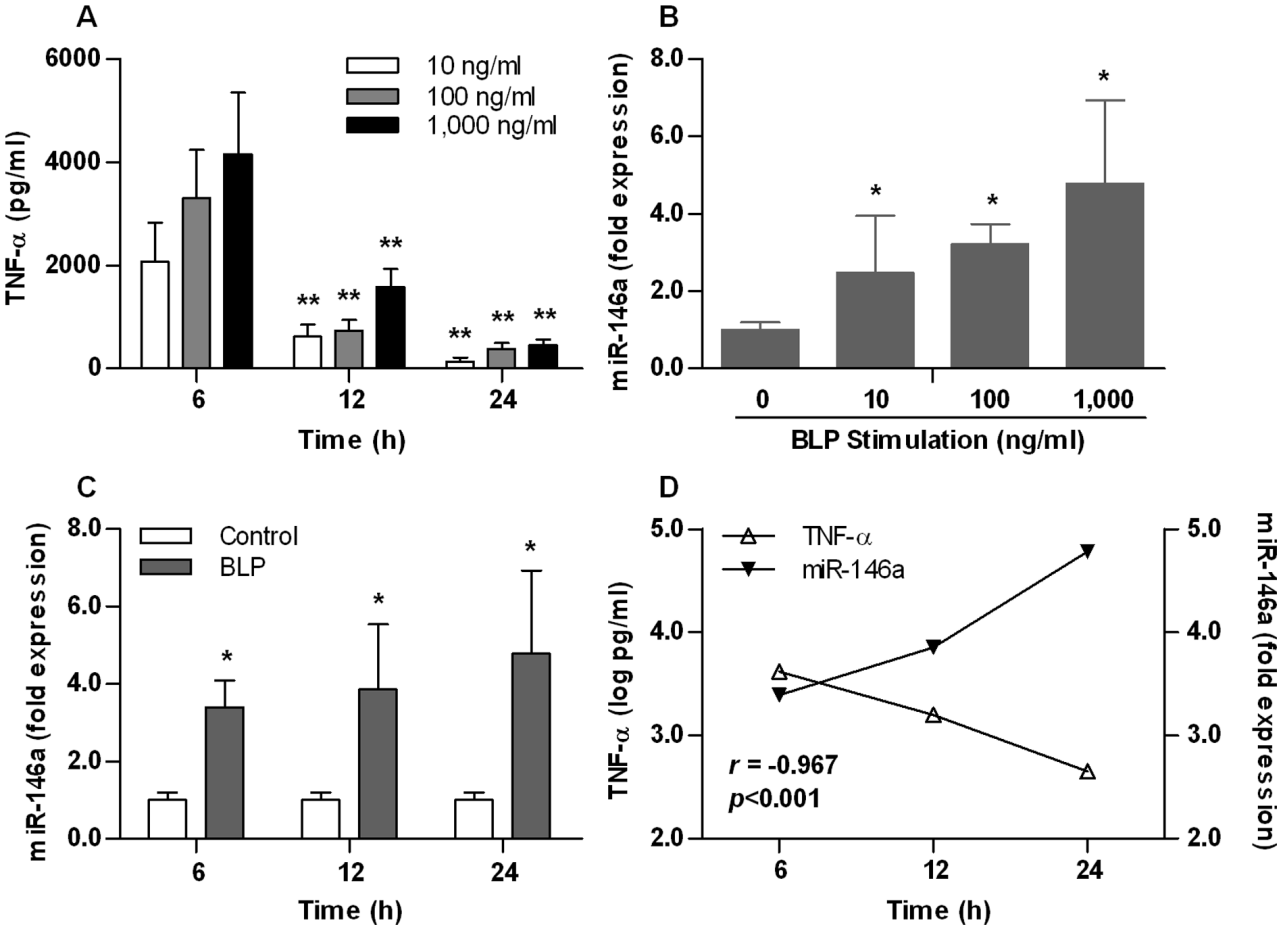
Activation of the TLR2 signaling upregulates miR-146a expression. (**A**) Human THP-1 cells were stimulated with various doses of BLP for 6, 12 and 24 h. (**B,** **C** and **D**) THP-1 cells were stimulated with various doses of BLP for 24 h (**B**) or stimulated with 1,000 ng/ml BLP for various time periods (**C** and **D**). THP-1 cells incubated with culture medium were used as the control. TNF-α concentrations in the culture supernatants were assessed by ELISA, and miR-146a levels in THP-1 cells were detected by real-time PCR and expressed as fold expression. Data are presented as mean ± SE of three independent experiments, and each experiment was conducted in triplicate. (**A,** **B** and **C**) *p<0.05, **p<0.01 compared with naive cells or the 6-h levels of either TNF-α or miR-146a. (**D**) The *r* value was calculated using the Pearson correlation coefficient test.

Human THP-1 cells were stimulated with variable doses of BLP at 10, 100 and 1,000 ng/ml for 6, 12 and 24 h. Maximal TNF-α levels were seen at 6 h with a fall in TNF-α levels at 24 h (*p*<0.01 versus the TNF-α levels at 6 h) and this occurred in a dose-dependent manner (Figure 1A). miR-146a expression was also induced by BLP stimulation in a dose-dependent manner (*p*<0.05 versus naive cells) (Figure 1B). Maximal miR-146a levels were seen with 1,000 ng/ml BLP after a 24-h stimulation (Figure 1C). As shown in Figure 1D, opposing responses were seen from TNF-α and miR-146a in relation to prolonged stimulation with BLP. Whilst TNF-α levels decreased with a longer duration of BLP stimulation, miR-146a expression levels continued to rise after 24 h of BLP stimulation, indicating a strong negative correlation between TNF-α levels and miR-146a expression in response to BLP stimulation (*r* = −0.967; *p*<0.001).

### miR-146a Plays a Role in BLP Tolerance

**Figure 2:**
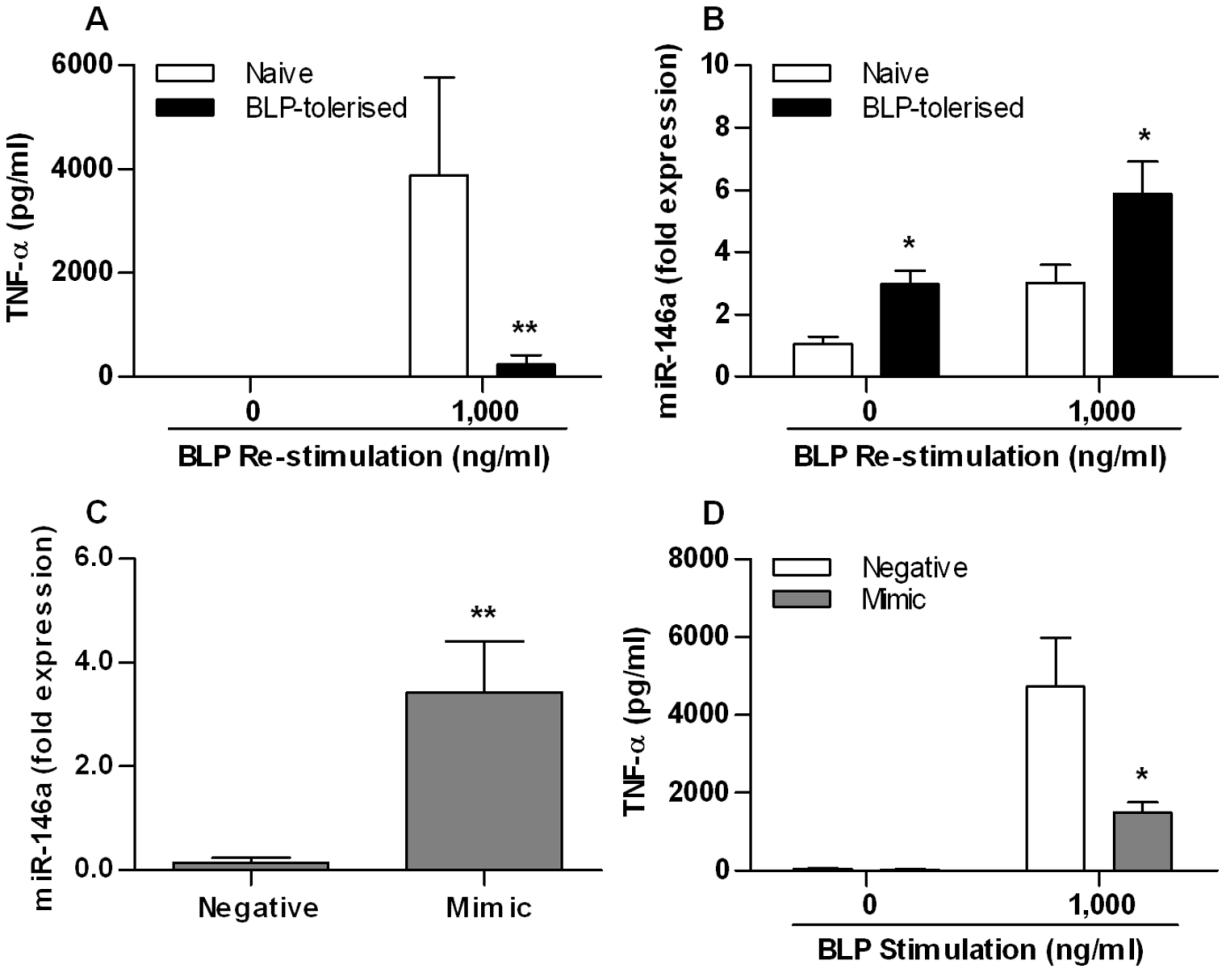
miR-146a is involved in BLP-induced self-tolerance. (**A** and **B**) Human THP-1 cells were pre-incubated with either culture medium (naive) or 100 ng/ml BLP (BLP-tolerised) for 18 h, and further stimulated with 1,000 ng/ml BLP for 6 h (**A**) or 24 h (**B**). (**C** and **D**) THP-1 cells were transfected with 40 nM of either miR-146a mimic or miRNA negative control (**C**) and then stimulated with 1,000 ng/ml BLP for 6 h (**D**). TNF-α concentrations in the culture supernatants were assessed by ELISA and miR-146a levels in THP-1 cells were detected by real-time PCR. Data are presented as mean ± SE of three independent experiments and each experiment was conducted in triplicate. *p<0.05, **p<0.01 compared with naive cells or miRNA negative control-transfected cells.

Naive and BLP-tolerised THP-1 cells were re-stimulated with 1,000 ng/ml BLP for 6 h. As shown in Figure 2A, BLP-tolerised cells showed a substantial reduction in TNF-α levels in response to BLP re-stimulation compared with naive cells (*p*<0.01). By contrast, miR-146a levels were significantly upregulated in BLP-tolerised cells following BLP re-stimulation (*p*<0.05 versus naive cells) (Figure 2B). Transfection of THP-1 cells with miR-146a mimic resulted in significant overexpression of miR-146a when compared to transfection with miRNA negative control (Figure 2C), confirming successful transfection of miR-146a mimic in THP-1 cells. Moreover, transfection of THP-1 cells with miR-146a mimic led to significant suppression of TNF-α in response to BLP stimulation compared with cells transfected with miRNA negative control (*p*<0.05) (Figure 2D).

### miR-146a Participates in BLP-induced Cross-tolerance to Gram-negative Bacteria

**Figure 3:**
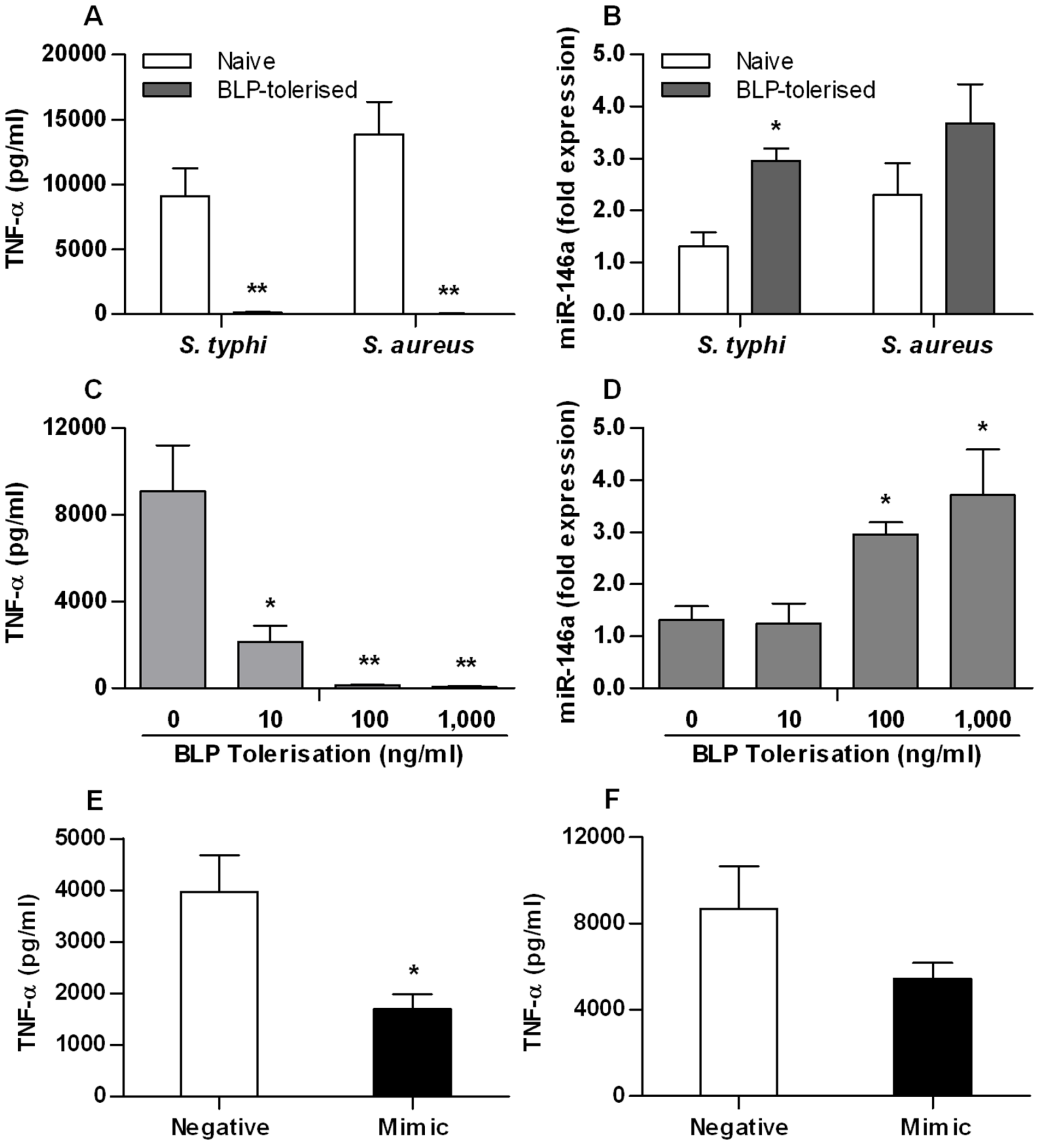
miR-146a participates in BLP-induced cross-tolerance to gram-negative bacteria. (**A** and **B**) Human THP-1 cells were pre-incubated with either culture medium (naive) or 100 ng/ml BLP (BLP-tolerised) for 18 h, and further stimulated with 1×10^6^ CFU/ml heat-killed *S. typhimurium* (*S. typhi*) or 1×10^7^ CFU/ml heat-killed *S. aureus* for 6 h. (**C** and **D**) THP-1 cells were pre-incubated with various doses of BLP for 18 h, and further stimulated with 1×10^6^ CFU/ml *S. typhimurium* for 6 h. (**E** and **F**) THP-1 cells were transfected with 40 nM of either miR-146a mimic or miRNA negative control and then stimulated with 1×10^6^ CFU/ml *S. typhimurium* (**E**) or 1×10^7^ CFU/ml *S. aureus* (**F**) for 6 h. TNF-α concentrations in the culture supernatants were assessed by ELISA, and miR-146a levels in THP-1 cells were detected by real-time PCR and expressed as fold expression. Data are presented as mean ± SE of three independent experiments, and each experiment was conducted in triplicate. *p<0.05, **p<0.01 compared with naive cells or miRNA negative control-transfected cells.

Naive and BLP-tolerised THP-1 cells were re-stimulated with either heat-killed *S. typhimurium* (1×10^6^ CFU/ml) or heat-killed *S. aureus* (1×10^7^ CFU/ml) for 6 h. As demonstrated in Figure 3A, the almost completely attenuated TNF-α release in the supernatants confirmed BLP-induced cross-tolerance to both gram-negative and gram-positive bacteria (*p*<0.01 versus naive cells). In BLP-tolerised cells, subsequent stimulation with gram-negative *S. typhimurium* caused significantly increased expression of miR-146a (*p*<0.05 versus naive cells) (Figure 3B). However, this was not the case in BLP-tolerised cells subsequently stimulated with gram-positive *S. aureus* (Figure 3B).

Dose-dependent tolerisation was then examined by pre-treating THP-1 cells with BLP at 10, 100 and 1,000 ng/ml for 18 h and re-stimulating these cells with heat-killed *S. typhimurium* for a further 6 h. BLP-induced cross-tolerisation to *S. typhimurium* was shown to be BLP dose-dependent, as confirmed by the attenuated TNF-α release (Figure 3C). *S. typhimurium*-induced upregulation of miR-146a expression in BLP-tolerised cells was also shown to be dependent on the tolerising dose of BLP (Figure 3D).

Finally, we transfected THP-1 cells with miR-146a mimic or miR negative control and re-stimulated these cells with either heat-killed *S. typhimurium* (1×10^6^ CFU/ml) or heat-killed *S. aureus* (1×10^7^ CFU/ml) for 6 h. In comparison to THP-1 cells transfected with miR negative control, miR-146a mimic transfection resulted in a significant attenuation in *S. typhimurium* stimulation-induced TNF-α production (p<0.05) (Figure 3E). By contrast, although transfection with miR-146a mimic led to partially reduced TNF-α release from cells stimulated with *S. aureus*, it did not reach a statistical significance (Figure 3F).

### Both IRAK-1 and Phosphorylated IκBα are Inhibited in miR-146a Mimic-transfected Cells

**Figure 4:**
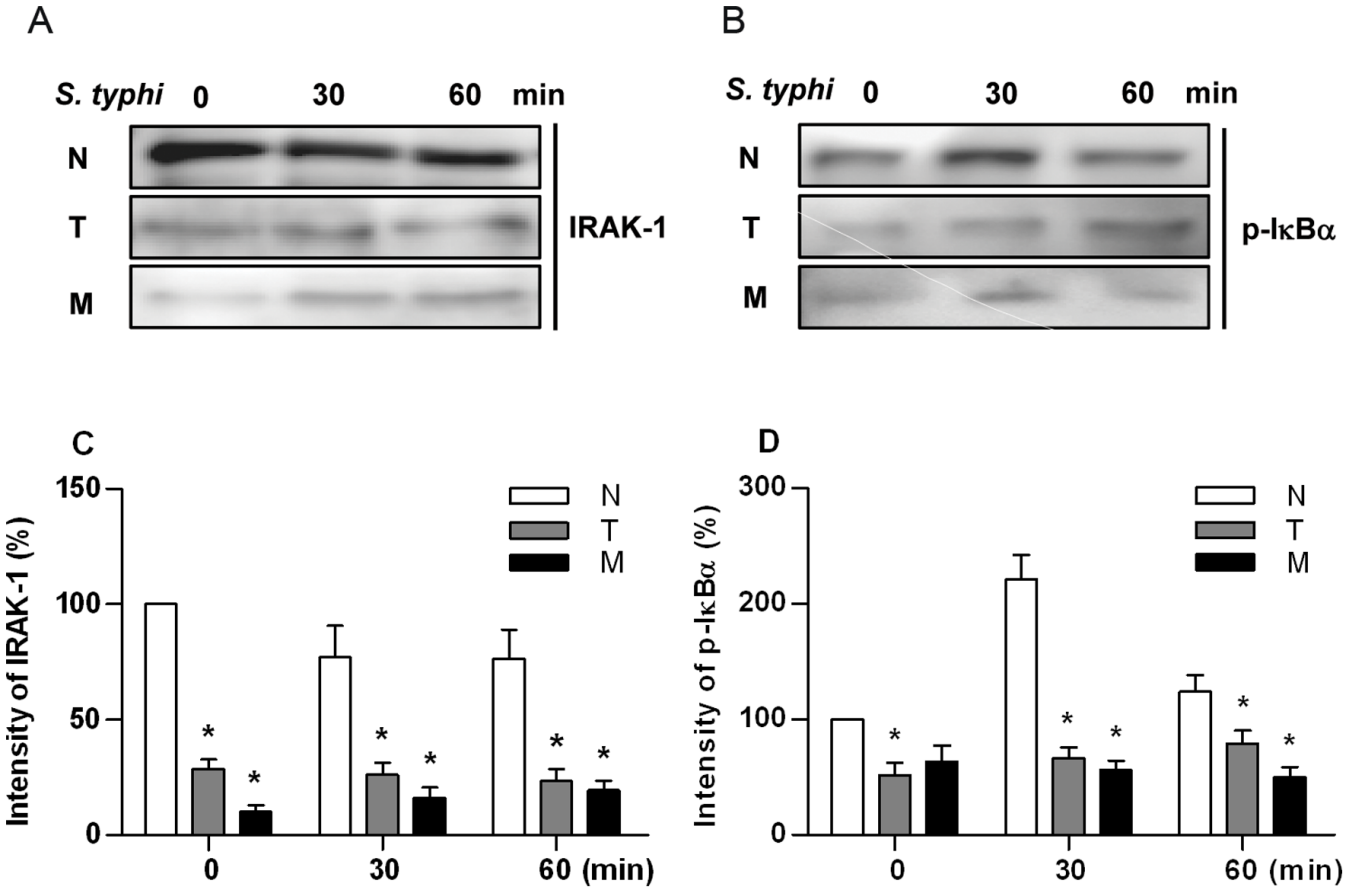
Overexpression of miRNA-146a attenuates IRAK-1 expression and IκBα phosphorylation. Human THP-1 cells were pre-incubated with either culture medium (naive) or 100 ng/ml BLP (BLP-tolerised) for 18 h, or transfected with 40 nM miR-146a mimic. Naive (N), BLP-tolerised (T) and miR-146a mimic-transfected (M) cells were further stimulated with 1×10^6^ CFU/ml heat-killed *S. typhimurium* for the indicated time periods. Cytoplasmic proteins were extracted and subjected to immunoblotting for detection of expression of IRAK-1 (**A**) and phosphotylated IκBα (p-IκBα) (**B**). The intensity of each band corresponding with the signal of either IRAK-1 (**C**) or p-IκBα (**D**) was corrected by its corresponding β-actin band after scanning and expressed as the percentage of the intensity detected in naive cells. All data are presented as mean ± SE of three separate experiments. *p<0.05 compared with naive cells.

Both reduced IRAK-1 protein expression and inhibited IκBα phosphorylation have been shown to be associated with BLP-induced tolerance [27]. Therefore, we assessed IRAK-1 and phosphorylated IκBα expression in naive, BLP-tolerised and miR-146a mimic-transfected THP-1 cells stimulated with heat-killed *S. typhimurium* (1×10^6^ CFU/ml) for various time periods. Stimulation of naive THP-1 cells with *S. typhimurium* led to a relatively reduced IRAK-1 expression (Figure 4A and 4C) and markedly enhanced IκBα phosphorylation 30 min post stimulation (Figure 4B and 4D), whereas BLP-tolerised cells displayed a strong inhibition in both IRAK-1 expression and IκBα phosphorylation in response to *S. typhimurium* stimulation (Figure 4A-D). Transfection of miR146a mimic in naïve THP-1 cells caused a substantial decrease in IRAK-1 expression (Figure 4A and 4C) and attenuated *S. typhimurium*-induced phosphorylation of IκBα (Figure 4B and 4D), similar to those seen in BLP-tolerised cells.

## Discussion

In the present study, we showed that BLP induced TNF-α production in THP-1 cells in a dose-dependent manner and that BLP also upregulated miR-146a expression in THP-1 cells as shown in prior studies [15], [26]. We have additionally confirmed that miR-146a upregulation increases over time with ongoing BLP exposure [26], and the addition of our 12-h timepoint showed that this occurs in a dose-dependent fashion. Our data have newly demonstrateed that BLP-induced miR-146a expression is dose-dependent and that the time-dependent rise in miR-146a levels correlates with the simultaneous decrease in TNF-α levels seen over time. This suggests that miR-146a may play a direct or indirect role in suppression of TNF-α expression, as has since been shown in studies with LPS [23], [29]. Our data also suggest that miR-146a may play a similar role in BLP-induced tolerance. We found that miR-146a was further upregulated in BLP-tolerised cells in response to BLP re-stimulation. Transfection of miR-146a mimics in naive cells led to reduced TNF-α release in response to BLP stimulation, a phenomenon which has previously been shown to also occur in response to LPS stimulation [23]. These results and ours suggest miR-146a may be instrumental in the development of bacterial components-induced tolerance.

Studies to determine the mechanism of action of miR-146a have shown that miR-146a targets and inhibits IRAK-1 and TRAF6 [15], [23], suggesting that miR-146a may play a negative feedback role on the MyD88-dependent pathway. El Gazzar et al. [29] demonstrated two further roles for miR-146a in development of endotoxin tolerance. Firstly miR-146a was shown to be involved in binding of the transcriptional repressor RelB to the TNF-α promoter region; miR-146a also regulates translational repression of TNF-α by preventing interaction of RNA-binding protein effectors argonaute-1 and RNA binding motif 4. However, the degree of tolerance induced by miR-146a overexpression alone did not reach the TNF-α suppression levels seen in BLP tolerance – in our study TNF-α levels were reduced from 4,731 pg/ml to 1,487 pg/ml in the miR-146a mimic-transfected group compared to reduction from 3,937 pg/ml to 232 pg/ml in the BLP-tolerised group. The only other study which has looked at miRNA in tolerance via the TLR2 signaling pathway also found that TNF-α suppression was not seen to the same extent in miR-146a mimic-transfected cells and PGN-tolerised cells [26]. Whilst miR-146a mimic transfection induces tolerance effects it does not completely reduce TNF-α expression, suggesting that miR-146a is not the solely responsible mediator for tolerance in these cells. Other miRNAs which are known to target TNF-α expression in LPS-tolerised cells are miR-221, miR-579 and miR-125b [30]. However, mimic transfection of miR-221, miR-579 and miR-125b only restores TNF-α levels to approximately 80% that of non-tolerised cells [30]. As all four miRNAs target TNF-α, there is likely a mutual requirement for their expression for the full effects of tolerance to be seen.

We assessed miR-146a levels in BLP-tolerised cells cross-stimulated with both gram-negative and gram-positive bacteria and found that miR-146a expression was further increased in BLP-tolerised cells following a subsequent bacterial stimulus, reaching a significant difference in the case of stimulation with gram-negative *S. typhi*murium. Furthermore, we have shown that this effect is dependent on the tolerising dose of BLP used. We subsequently demonstrated that transfection of miR-146a mimic substantially attenuated *S. typhimurium*-stimulated TNF-α production, indicating that overexpression of miR-146a may account, at least in part, for the observed cross-tolerance effect.

As discussed previously, miR-146a targets and suppresses IRAK-1 and TRAF6 [15], [26]. We further demonstrated that miR-146a overexpression suppressed baseline and *S. typhimurium*-stimulated IRAK-1 expression to that of BLP-tolerised cells and that miR-146a mimic transfection resulted in a strong inhibition in *S. typhimurium*-induced activation of IκBα similar to that seen in BLP-tolerised cells. Jurkin et al. [31] have shown that miR-146a overexpression in PGN-stimulated monocytic cells results in dose-dependently diminished downstream signaling with lower levels of IκBα phosphorylation and degradation, indicating impaired NF-κB signaling. Phosphorylated p38 levels are also reduced but phosphorylated ERK and TLR2 expression levels are unaffected [31]. miR-146a mimic-transduced cells show consistently lower percentages of nuclear p65-positive cells [31]. Our results combined with those from Jurkin et al. [31] demonstrate that miR-146a overexpression downregulates IRAK-1 expression with concomitant reduction in NF-κB signaling. Induction of miR-146a is known to be NF-κB dependent [15] and thus is a pivotal component of a negative feedback loop invoked by TLR signaling.

The clinical significance of tolerance remains an area of debate. Strong suppression of proinflammatory signals due to high tolerance levels may create a degree of host “immunosuppression” potentially leaving them susceptible to further microbial infection. Complete LPS hyporesponsiveness may increase susceptibility to overwhelming infection [32] and endotoxin tolerance states are associated with the increased mortality due to secondary infection [9], [10]. On the other hand, an appropriate tolerance response can protect the host from an overwhelming inflammatory response which may itself prove fatal. Induction of tolerance can render a patient less susceptible to the pathological effects of circulating endotoxin and thereby reduce the exaggerated immune response to microbial sepsis which may have deleterious consequences [32]. The effects of tolerance both *in vitro* and *in vivo* have been shown to be dose-dependent and recent studies further confirm similar dose-dependent expression of “tolerising” miRNAs *in vitro* [23], [33]. The degree of miRNA expression induced or suppressed appears to be directly proportional to the dose of the initial stimulus and may therefore be partially responsible for the clinical tolerance phenotype which results. The dose-response relationship may be the key to the clinical implications of tolerance [34].

Taken together, our results demonstrate that miR-146a is upregulated by and negatively regulates the TLR2 signaling pathway. These results suggest that miR-146a plays a role in BLP self-tolerance and cross-tolerance to gram-negative bacteria. miR-146a may represent a future target for exogenous modulation of tolerance in the host innate immune response to microbial infection.
